# A Systematic and a Scoping Review on the Psychometrics and Clinical Utility of the Volume-Viscosity Swallow Test (V-VST) in the Clinical Screening and Assessment of Oropharyngeal Dysphagia

**DOI:** 10.3390/foods10081900

**Published:** 2021-08-16

**Authors:** Stephanie A. Riera, Sergio Marin, Mateu Serra-Prat, Noemí Tomsen, Viridiana Arreola, Omar Ortega, Margaret Walshe, Pere Clavé

**Affiliations:** 1 Gastrointestinal Motility Laboratory, Hospital de Mataró, Consorci Sanitari del Maresme, 08304 Mataró, Catalunya, Spain; stephanie.riera@gmail.com (S.A.R.); smarin@csdm.cat (S.M.); ntomsen@csdm.cat (N.T.); oarreola@csdm.cat (V.A.); oortega@csdm.cat (O.O.); 2 Department of Surgery and Morphological Sciences, University Autonomous of Barcelona, 08193 Cerdanyola del Vallès, Catalunya, Spain; 3 Department of Pharmacy, Hospital Universitari Germans Trias i Pujol, 08916 Badalona, Catalunya, Spain; 4 Research Unit, Hospital de Mataró, Consorci Sanitari del Maresme, 08304 Mataró, Catalunya, Spain; mserra@csdm.cat; 5 Centro de Investigación Biomédica en Red de Enfermedades Hepáticas y Digestivas (CIBERehd), 08304 Mataró, Catalunya, Spain; 6 Department of Clinical Speech and Language Studies, Trinity College, 8PVW G5 Dublin, Ireland; walshema@tcd.ie

**Keywords:** oropharyngeal dysphagia, deglutition disorders, volume-viscosity swallow test, screening, diagnosis, sensitivity, specificity, V-VST, systematic review, swallowing disorders

## Abstract

(1) Background: The volume-viscosity swallow test (V-VST) is a clinical tool for screening and diagnosis of oropharyngeal dysphagia (OD). Our aims were to examine the clinical utility of the V-VST against videofluoroscopy (VFS) or fiberoptic endoscopic evaluation of swallow (FEES) and to map the V-VST usage with patients at risk of OD across the years since it was described for the first time, carrying a systematic and a scoping review. (2) Methods: We performed both a systematic review (SR) including studies that look at the diagnostic test accuracy, and a scoping review (ScR) with articles published from September 2008 to May 2020. Searches were done in different databases, including PubMed and EMBASE from September 2008 until May 2020, and no language restrictions were applied. A meta-analysis was done in the SR to assess the psychometric properties of the V-VST. Quality of studies was assessed by Dutch Cochrane, QUADAS, GRADE (SR), and STROBE (ScR) criteria. The SR protocol was registered on PROSPERO (registration: CRD42020136252). (3) Results: For the diagnostic accuracy SR: four studies were included. V-VST had a diagnostic sensitivity for OD of 93.17%, 81.39% specificity, and an inter-rater reliability Kappa = 0.77. Likelihood ratios (LHR) for OD were 0.08 (LHR–) and 5.01 (LHR+), and the diagnostic odds ratio for OD was 51.18. Quality of studies in SR was graded as high with low risk of bias. In the ScR: 34 studies were retrieved. They indicated that V-VST has been used internationally to assess OD’s prevalence and complications. (4) Conclusions: The V-VST has strong psychometric properties and valid endpoints for OD in different phenotypes of patients. Our results support its utility in the screening and clinical diagnosis and management of OD.

## 1. Introduction

Oropharyngeal dysphagia (OD) is a condition recognized by the World Health Organization (WHO), classified in the International Statistical Classification of Diseases and Related Health Problems with the codes ICD-9: 787.2 and ICD-10: R13 [1]. It is highly prevalent among several underlying pathologies and varies according to the severity of the concomitant disease, affecting up to 27% of community-dwelling older adults, over 50% of hospitalized or institutionalized older patients, 50% of stroke patients, from 24% to 86% of patients with neurodegenerative diseases, and from 17% to 86% of patients with head and neck structural disorders [2]. OD causes respiratory infections including aspiration pneumonia, which leads to mortality in up to 50% [3]. Other prevalent consequences are malnutrition and dehydration, with prevalences of 45% and almost 100% in older patients with OD, respectively [4]. One-year mortality of older patients with OD and malnutrition discharged from general hospitals is 65% [5].

The objective of early detection and treatment of OD is to avoid these severe respiratory and nutritional complications. The diagnostic algorithm of OD requires a three-step approach consisting of clinical screening and clinical and instrumental assessment [3]. Patients who have “failed” the screening test are at risk of OD and need further clinical and/or instrumental assessment. Despite its high prevalence and severe complications, OD is rarely systematically screened, and most hospitalized patients are not treated or even diagnosed. The goal of screening methods for OD should be to quickly identify patients at risk of OD who need clinical assessment [6]. In contrast, the goal of clinical assessment methods is to confirm OD and provide information on the specific impaired mechanisms of safety and efficacy of swallow to develop a therapeutic plan [3]. Finally, videofluoroscopy (VFS) and fiberoptic endoscopic evaluation of swallow (FEES) are the reference standard examinations to diagnose OD (instrumental assessment). However, it is not always possible to perform them on all patients at risk of OD [3].

**Figure 1:**
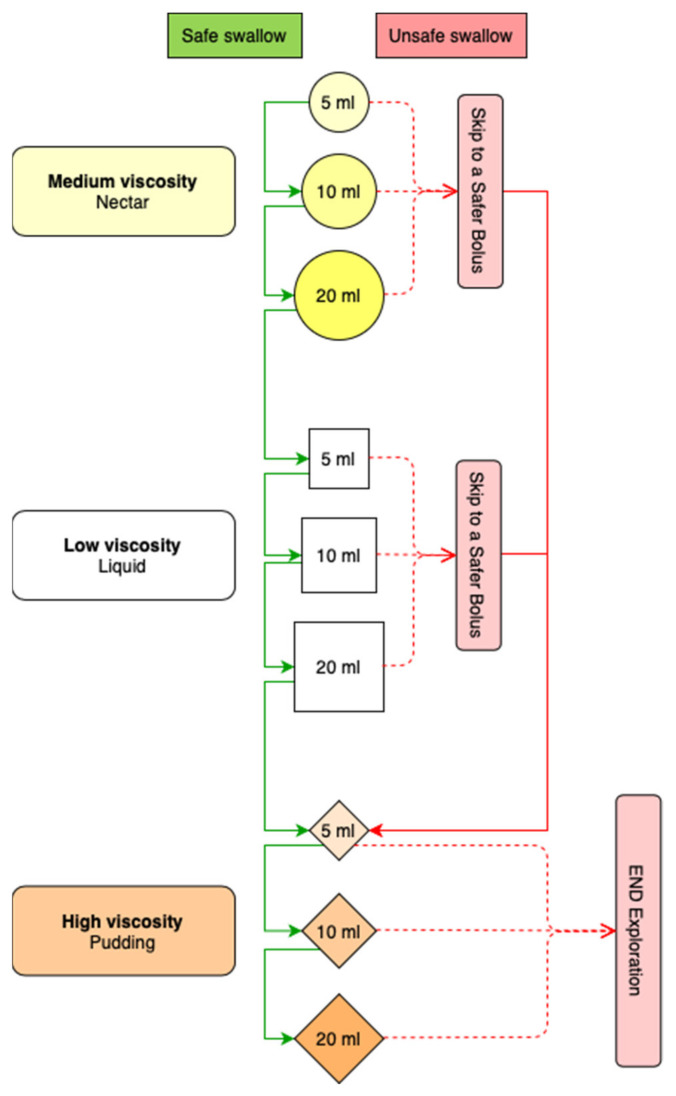
Algorithm of the volume-viscosity swallow test (V-VST). Patients with safe swallow start the test with a 5 mL medium bolus, followed by 10 and 20 mL, then perform with low viscosity following the same volumetric approach, and finally complete the test with high viscosity to explore the efficacy of swallow. If the patient presents any sign of impaired safety of swallow with any volume at medium or low viscosities, the procedure is interupted and the high viscosity is assessed. If there is any safety impairment with the high viscosity, the V-VST is ended. Green lines indicate a safe swallow, and red dashed lines indicate an unsafe swallow.

The volume-viscosity swallow test (V-VST) is a validated clinical tool that can be used to clinically assess OD and to provide accurate indications on the optimal bolus volume and viscosity for dysphagia patients. The V-VST was designed at the Hospital de Mataró, Catalonia, Spain, in 2005 to clinically screen and diagnose OD, and to assess the safety and efficacy signs of the swallow to indicate the bolus volume and viscosity level required as a compensatory measure for the optimal hydration of patients and to avoid complications [7]. The V-VST was designed to be a simple and cost-effective procedure; the only materials required are a syringe (50 mL capacity), thickener, water, and a pulse oximeter [7]. The V-VST is a swallowing effort test that evaluates the clinical signs of impaired efficacy (impaired labial seal, piecemeal deglutition, and oral and pharyngeal residue) and impaired safety of swallow (voice change, cough, and ≥3% oxygen desaturation measured with a pulse oximeter). The test was designed to use a series of three different bolus viscosities (medium—51–350 mPa-s, low—<50 mPa-s, and high—>1750 mPa-s) and three different bolus viscosity volumes (5 mL, 10 mL, and 20 mL) of increasing difficulty. The test starts with a trial of a 5 mL bolus of medium viscosity to reduce the risk for aspiration. If there are no signs (coughing, change in SpO2 levels, etc.) of impaired safety of swallow, the test continues by increasing volumes to 10 mL and 20 mL, and then using the same volume increments with the low- and then the high-viscosity bolus to assess efficacy. In contrast, if there are any signs of impaired safety, the test continues with the lower volume of the safer viscosity and terminates if a new impaired safety event occurs (Figure 1). The test is also useful because it allows the clinician to select the optimal bolus volume and viscosity to keep a safe and efficient swallow balance for each patient. Figure 1 shows the algorithm of progression of the V-VST [7].

The psychometric properties of V-VST were first published in 2008 [7], and since then it has been used extensively in different settings internationally in many phenotypes of patients with dysphagia [6,7,8,9]. In clinical practice, the V-VST is usually preceded by other patient-reported outcome-screening tools, such as EAT-10, to select patients at high risk for OD [6] and/or oromotor assessment; if the V-VST is positive, patients can be referred for instrumental examination (VFS, FEES) to further characterize the pathophysiology of the swallowing dysfunction and prescribe a specific treatment [7]. However, in some clinical settings, such as rehabilitation centres or nursing homes, or due to contexts such as the recent COVID-19 pandemic, it may not be feasible to perform an instrumental evaluation in every dysphagic patient, and the V-VST might become a very useful clinical tool for clinical assessment and clinical diagnosis of OD, and to help to maintain the optimal hydration status of dysphagic patients through fluid thickening [10].

Psychometric properties are characteristics of tests that describe and measure traits of an instrument, such as its diagnostic accuracy, validity, reliability, appropriateness, meaningfulness, and usefulness for a particular disease or condition [11]. V-VST psychometrics support it as a tool to detect patients at risk for dysphagia when used by nurses or general practitioners, hence in this case we describe it as an easy, quick, and accurate screening tool [9]. In addition, when used by a trained healthcare professional, this tool can be used as a clinical diagnostic tool for OD. In this case, the clinician will pursue more than just assessing the risk for OD, as they will be able to interpret the clinical signs of impaired safety and/or efficacy of swallow provided by the V-VST, and therefore the pathophysiology of OD, and also determine the optimal bolus volume and viscosity for a safe and efficient swallow provided by the test to formulate a tailored recommendation [6].

Our hypothesis in the SR was that the V-VST has strong psychometric values as a clinical test to clinically diagnose OD. No hypothesis was stated for the scoping review as per convention for scoping reviews; hypotheses were generated rather than tested in ScR.

The aims of this study were to evaluate the psychometric properties of the V-VST in the screening and diagnosis of OD, to evaluate its role in detecting impaired efficacy and impaired safety of swallow, and to map V-VST utility and usage reported in the literature since 2008, when the test was first described [7]. To address the aims of the study, we chose to combine two methodologies. A systematic review was performed to summarize, with rigorous methodology, the psychometrics of the V-VST evidenced scientifically. In order to complete the assessment of all available information in the literature on the use, scope around the world, and clinical applicability of V-VST, we completed this information with a scoping review so that a full picture of the test utility was collected in one single publication.

## 2. Materials and Methods

### 2.1. Protocol for the SR and ScR

For the SR, we followed the PRISMA methodology and included all studies that used or assessed psychometric properties of the V-VST. The ScR was completed using “The Scoping Review Checklist” from Cooper et al. [12], and all included studies were those that used the V-VST to assess the prevalence of OD, evaluate the complications of OD, estimate risk factors for OD, or examine outcomes of a study intervention, or papers that used V-VST in a clinical guideline.

### 2.2. Systematic Review (SR)

A SR protocol was registered in the International Prospective Register of Systematic Reviews produced by the Centre for Reviews and Dissemination (PROSPERO) with the number CRD42020136252 [13].

#### 2.2.1. Search Strategy

The search strategy was applied to 7 electronic databases: PubMed Central (PMC), MEDLINE using PubMed, EMBASE using Ovid, CINAHL, Web of Science, Cochrane, and Epistemonikos. This search was restricted by year, starting with papers published from September 2008 (when the instrument was first described) to November 2020. No language restriction was imposed.

A three-arm strategy combining the terms in each line with the Boolean “or” and between them with the Boolean “and” was used. Search terms were related to “OD”, “diagnosis”, and “V-VST” (Table 1). Reference lists of all included studies were carefully scrutinised to retrieve any additional eligible studies. The inclusion criteria were: (i) all studies that validated the V-VST as a clinical screening or diagnostic tool, regardless of underlying patients’ pathologies (e.g., neurodegenerative disease, post-stroke patients, elderly, head and neck diseases or anatomical alterations, and even healthy subjects); and (ii) use of V-VST with a reference test (VFSS or FEES). The exclusion criteria were: (i) studies not related to OD; (ii) studies related to oesophageal dysphagia; (iii) studies that did not include data on the V-VST; (iv) conference abstracts that included posters, textbooks, unpublished material; and (v) non-original studies.

#### 2.2.2. Selection Process

**Figure 2:**
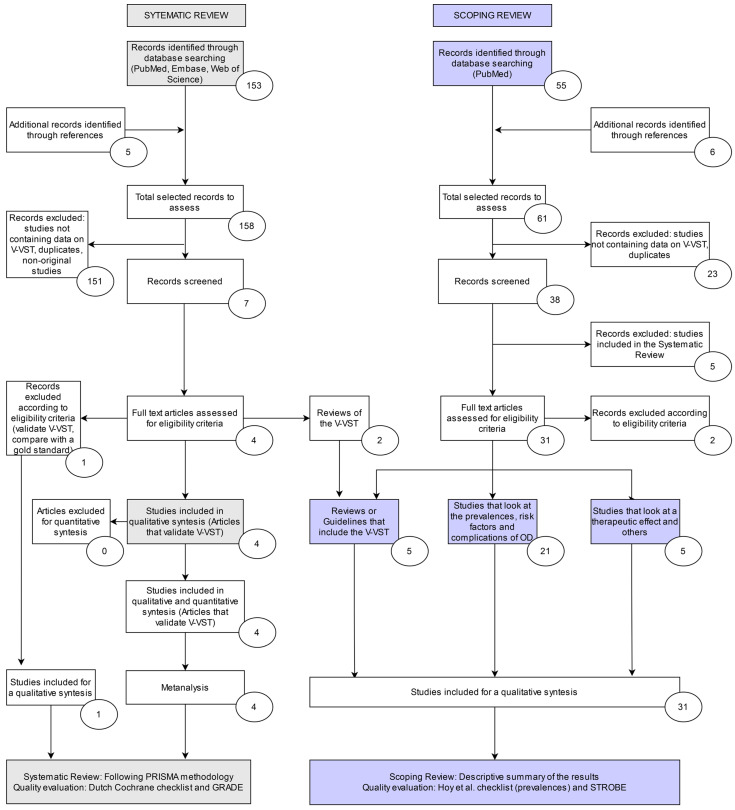
Systematic review (SR) and scoping review (ScR) flowcharts following the PRISMA guidelines [14,15] showing the selection process for the articles finally included in the SR (*n* = 5) and the ScR (*n* = 32).

After duplicates were removed, the selection process was conducted (see Figure 2) in two phases: an initial screening phase to determine eligibility of the study for inclusion in the SR, and a second phase to identify potential studies of interest for the ScR. After collecting all studies from the different resources in which the search was applied, duplicates were removed. The initial screening consisted of evaluating the title and abstract of each study. This process was performed independently by each reviewer; consensus for inclusion was achieved with no need for a third reviewer to mediate the final selection.

#### 2.2.3. Data Presentation, Summary Measures, and Data Analysis

Data extraction from selected studies was performed by two independent reviewers, and in case of disagreement, a third reviewer participated to reach a final consensus decision. Authors of included studies were contacted in case of missing data or to clarify possible items. Data was extracted using specifically devised data-collection forms We obtained the following data from each included study: (a) study identification: first author, year, title; (b) design characteristics: study arm and/or presence of a control group (non-OD affected or suspected patients), setting, time frame, and location; (c) study sample characteristics: sample size, sociodemographic data (age, gender), eligibility criteria, OD aetiology, prevalence by underlying pathology; (d) test characteristics: thickeners and viscosities used, methodology of the test, reference test; (e) psychometric characteristics: known prevalence of dysphagia, sensitivity (Se), specificity (Sp), positive predictive value (PPV), negative predictive value (NPV), positive likelihood ratio (LHR+), negative likelihood ratio (LHR–), diagnostic odds ratio (DOR); (f) evaluated endpoints: oropharyngeal dysphagia, impaired safety, impaired efficacy; (g) signs of IS: aspiration, penetration, cough, voice change, O2 desaturation; (h) signs of IE: oral residue, pharyngeal residue, piecemeal deglutition.

Psychometric properties of the test (Se and Sp) were combined regarding the endpoint analysed (OD and all V-VST endpoints related to impaired safety and efficacy of swallow). Study authors were contacted as required in order to obtain raw data if not available in the published study in order to perform a meta-analysis to reach a statistical conclusion for the included studies, where applicable. For those studies with sufficient data of the same endpoint, heterogeneity between studies was assessed using the Q statistics test. Homogeneity between studies was confirmed. Meta-analysis when appropriate was performed using a fixed-effect model for OD and impaired safety endpoints [16]. Confidence intervals (CI) (95%) for Se and Sp were calculated following the formula to calculate CI of proportions and taking into account the total number of subjects with confirmed OD for the sensitivity and total number of subjects without OD for the specificity. When disaggregated data could not be obtained, meta-analysis was not possible (this is the case for impaired efficacy and signs of impaired safety and impaired efficacy), and data was presented as ranges of Se and Sp and the simple mean of psychometric properties. DOR was calculated, and LHR+ and LHR– were calculated from the Se and Sp for each endpoint [14,17]. Fagan’s nomograms [18] represent the LHRs for OD, aspiration, impaired safety and impaired efficacy (Figure S1); calculations were performed based on an assumed estimate pre-test prevalence of OD 10% (community), 50% (hospitalized), and 75% (nursing homes).

Data are summarized in tables and nomograms. A narrative description and a more global comparison of the results is also presented to explain the salient findings and conclusions for outcomes of the test that could not be included in the meta-analysis.

#### 2.2.4. Quality Evaluation and Strength of the Evidence

To assess the methodological quality of the included studies, the “Criteria for methodological quality assessment of studies” (Dutch Cochrane Centre) and QUADAS-2 [19,20] were applied. The “Criteria for methodological quality assessment of studies” consists of nine items classified in the following manner: the first six items evaluate the validity of the study, the seventh examines generalizability, and the last two look at the reliability of the study. Each item was scored with “yes”, “no”, or “unclear”, achieving a total score that rated if the quality of the study was sufficient or not. “Yes” was given if the item had been addressed, “no” if it had not been addressed, and “unclear” if the item was not clear or partially available. Quality of a study was considered “sufficient” if a maximum of one item was “no” or “unclear”. QUADAS-2 is the current version of QUADAS and the tool that Cochrane recommends for use in systematic reviews to evaluate the risk of bias and applicability of primary diagnostic accuracy studies. QUADAS-2 consists of four key domains: (1) patient selection, (2) index test, (3) reference standard, and (4) flow and timing. Each is assessed in terms of risk of bias, and the first three in terms of concerns regarding applicability. Signalling questions are included to assist in judgements about risk of bias. QUADAS-2 is applied in four phases: (a) summarise the review question, (b) tailor the tool to the review and produce review-specific guidance, (c) construct a flow diagram for the primary study, and (d) assess risk of bias and concerns regarding applicability. If there were any missing or unclear data that could lead to confusion, after discussion with the other reviewer, authors of the original study were contacted. There was no plan for assumptions or data simplification, otherwise this would have been reported. The strength of evidence across studies was rated using GRADE (Grading of Recommendations Assessment, Development, and Evaluation) methodology [21], implying a GRADE diagnostic question for each endpoint evaluated (OD, IS, impaired efficacy, and aspirations) and using the option-pooled studies that combined the data of all studies included. In this SR, our hypothesis was that V-VST had strong psychometric values as a clinical test to diagnose OD, which it accomplished across different underlying pathologies.

### 2.3. Scoping Review (ScR)

The ScR followed the “Scoping Review Checklist” from Cooper et al. [12,22] and included all studies that used the V-VST for: (a) reviews or guidelines that include the V-VST as a clinical screening or diagnostic tool for OD; (b) studies on the nutritional or respiratory complications of OD, and studies that used the V-VST as the test to assess OD to define the prevalence or risk factors of OD in a specific phenotype or cohort of patients; (c) studies that applied the V-VST in the prescription for thickening fluids or to assess the outcome of an intervention; and (d) and other studies that used the V-VST and yet did not fit into any of the previous categories.

#### 2.3.1. Search Strategy

We used a wider strategy in MEDLINE using PubMed, with one only arm based on the V-VST, and including free terms combined between them with the Boolean “OR” (“Volume viscosity Swallowing test”) or (“V-VST”) or (“volume-viscosity swallow test”). This search was restricted by year, including publications from September 2008 until November 2020.

#### 2.3.2. Eligibility Criteria

Inclusion criteria were all studies that involved V-VST, such as: (1) reviews, systematic reviews, and guidelines on the V-VST (diagnosis, assessment, clinical utility, management); (2) prevalence, complications, and risk-factor studies using the V-VST; (3) therapeutic effect assessed with the V-VST; and (4) other studies using the V-VST that could not be included in the previous categories.

#### 2.3.3. Selection Process

The selection process was divided into two phases. The purpose of the initial phase was to exclude studies that did not include the V-VST and any duplicates, and consisted of the review of titles and abstracts; the second phase consisted of the analysis of the full text of the studies selected by the initial phase, to ensure they fulfilled the inclusion and exclusion criteria.

The review team consisted of three reviewers S.A.R., S.M., and O.O. All papers were screened individually by two reviewers blinded to the other’s selection. In case of discrepancy, the third reviewer was available to help reach a consensus. As in the SR, there were no sample-size restrictions.

#### 2.3.4. Data Presentation, Summary Measures, and Data Analysis

Data obtained in their original form were reported with narrative description and summarized in tables. Results are presented according to the following order: (i) underlying pathology, (ii) target population, and (iii) prevalence. A summary of the methods to perform the V-VST described in all the studies was included to report the following information: (a) thickening agents and levels of viscosities used (methodology is given in the Supplementary Materials); (b) methodology and endpoints—signs of impaired safety or impaired efficacy of swallow; and (c) information obtained regarding the optimal volume and viscosity for each patient. A table with all studies included, classified by the year and country of publication, helped to map the usability of the test reported since the first publication in 2008. In contrast to the SR, it was not possible to conduct a meta-analysis.

#### 2.3.5. Quality of Reporting and Strength of the Evidence

To provide a measurement of quality of reporting that covered all methodologies, we used the combined checklist of the STROBE (STrengthening and Reporting of OBservational studies in Epidemiology) guidelines for the recommendations on what should be included in an accurate and complete report of an observational study, and calculated the percentage of accomplished points (of those that each study could apply) of included cohort studies, cross-sectional studies, and case-control studies included [23]. In addition, the quality of prevalence studies was assessed using the “Quality assessment checklist for prevalence studies” [24,25].

## 3. Results

### 3.1. Systematic Review (SR)

#### 3.1.1. Studies Included

The results of the bibliographic database searches identified 155 articles. Five additional studies were identified by checking bibliographic references. A total of 160 articles were screened by the reviewers (Figure 2). After the screening phase, just seven were selected to be included in the selection phase, and 153 were excluded since they did not meet the criteria for using or validating the V-VST after revising the title and abstract. After reviewing the full text, two more were excluded for not meeting the eligibility criteria because they were reviews [2,3]. One was excluded for not comparing the V-VST with a reference test such as the VFS or FEES [26]. These four studies assessed psychometric properties of the V-VST test in the diagnosis of OD in different phenotypes of patients. Clavé et al. [7] validated the test in 2008 including 97 participants: 85 patients at risk of dysphagia with a range of underlying pathologies (elderly, neurodegenerative diseases, and head and neck diseases), and a control group of 12 healthy volunteers. The study was performed by trained healthcare professionals and validated against VFS as “reference standard”. In 2012, Paris et al. [8] validated the V-VST vs. VFS with a cohort of 20 patients with ALS. In 2013, Guillén-Solà et al. [9] validated the V-VST vs. VFS in 52 sub-acute stroke patients admitted to a rehabilitation unit (Table 2). Finally, Rofes et al. [6] validated the V-VST against VFS using a control group of healthy volunteers and a variety of participants at risk of OD, including elderly, stroke, and neurodegenerative disease patients, with a total of 134 participants.

#### 3.1.2. VST Psychometrics: Se and SP, Likelihood Ratio, and Odds Ratio

Table 2 summarizes the psychometric values reported in each study. The main results weighting the data from the different publications, combined with meta-analysis, showed an overall 93.17% Se and 81.39% Sp for the clinical diagnosis of OD [6,8]; and 86.07% Se and 68.47% Sp for the clinical diagnosis of impaired safety of swallow [7,8,9]. LHR– for OD was 0.08 and LHR+ was 5.01, and for impaired safety, 0.20 (LHR–) and 2.73 (LHR+). DOR for OD was 51.18 (95% CI 15.29–171.32), and for impaired safety, 11.67 (95% CI 5.47–24.92). Results summarizing the psychometric properties regarding aspiration, impaired efficacy of swallow, and other signs of OD are presented in Table 3. Nomograms showed the effect on post-test probability of OD, aspiration, impaired safety, and impaired efficacy of swallow according to the LHR+ and LHR– of the test (Figure S1).

#### 3.1.3. Reliability of the V-VST

Reliability of the test was only described in one of the included articles of the SR [6]; it showed an inter-observer reliability with a kappa value of 0.628 (95% CI 0.45–0.78). However, there was another article included in the ScR [26] that looked at the inter-rater reliability between three different hospitals in Denmark in a geriatric population admitted to an acute care centre. Eleven skilled occupational therapists performed the test, blinded, within a maximum difference of one hour between raters. The overall Kappa value was 0.77 (95% CI 0.65–0.89); however, the inter-rater reliability differed among hospitals, ranging from 0.37 (95%, CI 0.01–0.41) to 0.85 (95%, CI 0.75–1.00). 

#### 3.1.4. The V-VST as a Tool for Therapeutic Recommendations on Bolus Modification

An Se of 84.6% and an Sp of 73.4% was reported for the V-VST in selecting patients with swallow-safety improvement by increasing bolus viscosity [7]. They also reported a significant improvement in safety of swallow and a reduction in prevalence of penetrations and aspirations caused by increasing viscosity, and a significant safety impairment as the bolus volume was augmented for all viscosities [7]. Paris et al. highlighted the advantage of the test to indicate a volume and viscosity for each patient, to avoid disturbances of the propulsion (efficacy) and laryngeal aspiration or bronchial penetration (safety) [8]. Guillén-Solà et al. outlined that the V-VST allows early dietary adjustments by detecting the volumes and viscosities for which the patient is at risk [9]. Rofes et al. found that 72.40% of the participants had improved safety of their swallow by increasing bolus viscosity [6].

#### 3.1.5. Quality of Studies in the SR

Quality assessment of all studies validating the V-VST included in the SR found a low risk of bias according to QUADAS-2 [27], and sufficient quality according to the Dutch Cochrane Quality Assessment [19]. The strength of the evidence was found as high using GRADE for all test endpoints (OD, impaired safety, impaired efficacy, and aspirations) (Table 4) [21].

### 3.2. Scoping Review (ScR)

#### 3.2.1. Studies Included

A total of 62 articles were initially screened by the reviewers (55 articles from a PubMed search and seven from the screening of bibliographic references). From this initial screening, 39 articles were eligible for evaluation in the selection phase of the reviewer, including the five articles that formed the SR. Based on this selection process, two articles were excluded according to the eligibility criteria, and 32 articles were then classified into the three subgroups below (three papers were included in two categories).

Reviews and guidelines. Five studies from four different countries (Spain (2), the Netherlands (1), China (1), and the UK (1)) were included in this subgroup. This section included two SRs, a review of the V-VST, a guide on the management of OD in patients with advanced dementia, and a study that compared the V-VST with the water-swallow test (WST) in acute stroke patients. General conclusions of the papers stated that the V-VST is one of the bedside screening methods with the highest sensitivity and specificity for clinical diagnosis of OD, is simple and fast to perform (5–10 min), provides information about dysphagia severity, and can be used in any healthcare setting. In addition, the V-VST provides information on the most suitable bolus (volume and viscosity) for each patient, and thus it is useful in helping to maintain the hydration status of patients through fluid thickening.

Prevalence, complications, and risk factors. This section included 22 studies from six different countries (Spain (14), Denmark (3), Brazil (2), France (1), the Netherlands (1), and China (1)) that used the V-VST as the clinical assessment tool to either diagnose OD in order to assess its prevalence, or to evaluate the complications and risk factors of OD. Prevalence rates of OD found among the mentioned publications are summarized in Table 3. General conclusions of the revised papers stated that prevalence of OD is very high in different phenotypes of patients with OD (stroke, patients with neurodegenerative diseases, and older patients), and that OD is a risk factor for the development of severe complications such as malnutrition, dehydration, lower respiratory tract infections, community-acquired pneumonia, frailty, poor outcomes, and mortality. The papers also investigated the physiopathology of OD of specific phenotypes such as post-stroke patients, and recommended a universal screening of OD with the V-VST in at-risk populations.

Therapeutic effect and miscellaneous. This subgroup included eight studies from four different countries (Spain (3), China (1), Chile (1), and Denmark (3)) that evaluated the efficacy of a therapeutic treatment using the V-VST to assess OD severity pre- and post-treatment, or for other reasons (e.g., as an inclusion measure or to check the cost of OD), including one specific study that examined the inter-rater reliability of the V-VST. General conclusions from the articles included were that the effect of thickeners on the treatment of OD was viscosity-dependent, meaning that the higher the viscosity, the lower the prevalence of unsafe swallows; that OD was associated with low levels of SP; that pharmacological stimulation improved swallowing function in stroke patients with OD; that the EAT-10 questionnaire was a valid and reliable screening test, and that OD was associated with higher healthcare cost in the geriatric population.

The content and main conclusions of all publications included in the ScR are summarized in Table S1.

#### 3.2.2. STROBE Report on the Studies in the ScR

According to STROBE (24), all the studies included in the ScR achieved over 70% of item completion, meaning that they had a sufficient quality. In addition, all prevalence studies had a low risk of bias according to the Hoy et al. checklist (scores equal to or below 3) (Table 4) [25,58].

#### 3.2.3. Methods to Perform the V-VST in the Studies Included in the SR and the ScR

Thickening agents and viscosities used. Initial validation studies were performed with a starch-based thickener (Resource ThickenUp, Nestlé Nutrition, Barcelona, Spain) following the algorithm defined in 2008 (7) that used liquid viscosity (water) and two viscosity levels described by using the qualitative descriptors “nectar” and “pudding”, which correspond to 250–295 mPa-s and 3500–3900 mPa-s, respectively, according to the published viscosities. All studies included in the SR used either Resource ThickenUp [7,8,9] or Resource ThickenUp Clear [6,26] (Nestlé Nutrition, Barcelona, Spain). For the latter thickener, the descriptors were “nectar” and “extreme spoon-thick”, and the viscosities were 238 mPa-s and 1840 mPa-s, respectively [6]. Regarding the ScR, we found information on the thickener used in 51.61% of the included articles (16/31), with a majority of studies using the starch-based thickener Resource ThickenUp (15/16). Viscosity levels were only described in four of the articles. The main descriptors used were “nectar” for the intermediate viscosity and “pudding” or “spoon-thick” for the highest viscosity with the starch-based thickener; and “nectar” or “extreme spoon-thick” with the xanthan-gum-based thickener (Resource ThickenUp Clear). A summary of all the descriptors used and their specific shear viscosities measured at 50s-1 in International System units (mPa-s) is depicted in Table S2. Boluses were administered with a syringe in almost all studies to provide an accurate bolus volume to the patient, and boluses were delivered in the anterior part of the mouth following the originally described algorithm, with a bolus of 5, 10, and 20 mL for each viscosity series (Figure 1). However, in the ScR, we found two articles that studied OD in older patients with advanced dementia that modified the V-VST according to their participants’ limitations. They used a teaspoon instead of a syringe, and began with a smaller volume of 2.5 mL and a maximum bolus volume of 15 mL.

Geographical data. The V-VST has been used in studies published in eight different countries distributed among South America, Europe, and Asia (Table 4) F.

## 4. Discussion

This review showed that more than a decade from its description and initial validation by P. Clavé et al. [7], the V-VST is now used internationally for clinical screening and clinical diagnosis of OD, to select the most appropriate bolus volume and viscosity in dysphagic patients, to determine the prevalence of the condition, and to assess the clinical outcome and the effect of treatments applied to dysphagic patients. The two reviews included in this manuscript showed very good psychometric properties of the V-VST for OD and impaired safety and efficacy of swallow, and good reliability when applied by trained and experienced professionals.

### 4.1. Systematic Review (SR)

There are four main psychometric properties that characterize the V-VST and support its high clinical utility in the clinical screening of OD: its high diagnostic sensitivity to OD (93.17%) and low negative LHR (0.08), its high diagnostic odds ratio (51.18), and its good inter-rater reliability (Kappa = 0.77). The first psychometric quality of the V-VST is its very high sensitivity, meaning that up to 93% patients with OD will be identified by the test [59]. A high sensitivity is clearly important when the test is used to identify a serious but treatable disease such as OD or aspiration. The second psychometric quality is its strong negative LHR of 0.08 (LHR– is the probability of a negative test corresponding to a person who does not have the disease). A negative LHR below 0.1 usually is considered good. It depicts a very large decrease (10-fold) in post-test probability of disease following a negative test, and is not affected by prevalence. The high diagnostic OR of the V-VST is defined as the ratio of the odds of the test being positive if the patient has a disease relative to the odds of the test being positive if the patient does not have the disease (14). Higher diagnostic ORs are indicative of better test performance. A DOR > 25 can be considered as a good test. The DOR of the V-VST for OD was 51.18. Finally, a Kappa value of up to 0.77 can also be considered indicative of a very reliable test when applied by trained and experienced professionals [60]. Taking all these properties together, our SR described the V-VST as a very good tool for clinical screening of OD, impaired safety, and aspirations. Failing to detect OD may lead to severe complications that are associated with re-hospitalizations and increased mortality. In addition, a very low LHR– allows the disease to be excluded in patients with a negative test.

However, it is also important to highlight that the V-VST had a specificity of around 80% and a relatively poor LHR+, indicating a high rate of false positives and a low positive predictive value when the prevalence of OD was low. These data suggest that the V-VST is a good tool to rule out OD, but not to confirm it, meaning it is a good screening instrument, but not a good diagnostic test when the prevalence of the disease is low, such as in primary care. On the contrary, when the prevalence of the disease is very high (very-high-risk populations include those in geriatric units or stroke units of hospitals or nursing homes), the false positive rate will greatly improve, and the V-VST could be considered a valid clinical diagnostic test. In summary, the V-VST can be considered as having very good sensitivity and an acceptable specificity. Its clinical utility as a screening test or as a diagnostic test depends fundamentally on the prevalence of OD. Thus, in the context of primary care or in the hospital setting with a prevalence of no more than 30%, this test had a positive predictive value of 68.3% (31.7% false positives) and a negative predictive value of 96.6% (4.4% false negatives), indicating that it can be a good screening test, but not a good diagnostic test, in these scenarios. On the other hand, in geriatric or stroke units of acute hospitals, or in nursing home settings or in high-risk populations with a prevalence of OD that reaches 80%, the positive predictive value reaches 95.2%, and the negative predictive value 74.8%. In these contexts, the V-VST can be considered a useful clinical diagnostic test, especially given the low clinical relevance of the consequences of a false positive in these populations (treating of OD in a person without dysphagia). Thus, the V-VST can be considered a screening test or a clinical diagnostic test, depending on the prevalence of OD in the context or clinical setting in which it is applied.

The V-VST’s psychometrics are better than those of several other tests used to screen for OD. Water tests are the most frequently used, in which patients are asked to swallow a large amount of water (variable according to the test used) without interruption, and an alteration is identified by coughing during or after swallowing, wet voice quality, or slow swallowing (<10 mL/s) [61]. Bours et al. recommended in their systematic review to use a water test with pulse oximetry, with cough, choking, and voice changes as endpoints [57]. However, water tests do not assess the efficacy of the swallow or evaluate the ability to swallow different viscosities, putting the patients at high risk of aspirations, as they involve swallowing of quite large amounts of water without interruption. Other tests are more similar to the V-VST, testing different consistencies to evaluate aspiration and/or penetration. Sensitivities of these tests ranged from 41 to 100%, and specificity from 57 to 82% [62]. Kertscher et al. (2013) [32] offered an overview on the main bedside screening tools (V-VST, 3 oz water-swallow tests, trial swallow with water, TOR-BSST, and cough elicitation) to detect OD in neurological patients. They stated that in order to choose the screening tool, psychometrics and work-setting limitations need to be taken into account. In their study, they evaluated a total of four tests, but only two showed reliable psychometrics: the V-VST (95.8% Se and 63.0% Sp) and the TOR-BSST (91.3% Se and 66.7% Sp) [63]. These authors concluded that the V-VST is a valid and reliable screening tool for OD that includes oximetry and helps to select the safest viscosity for a patient’s oral intake. A recent meta-analysis showed 96.0% Se and 65.0% Sp for the Gugging Swallowing Screen [27], but this test and the TOR-BSST only have been validated to screen swallowing post-stroke. A clinical bedside test for OD should present good psychometric properties and reliability, but also a detailed and easy-to-perform protocol to protect patients’ safety and with the ability to evaluate the efficacy and safety of swallow, including the detection of silent aspirations, in different phenotypes of patients. All these conditions are fulfilled by the V-VST.

### 4.2. Scoping Review (ScR)

In the ScR, we found that the V-VST has been used internationally (eight different countries on three continents) and for several purposes, such as to determine the prevalence of OD or to assess the effect of treatments. A total of 34 studies from 2008 to 2020 were included according to our criteria, showing that the V-VST has been used in patients in a broad range of ages (but always adults), underlying diseases, and purposes across the world. Authors have chosen the V-VST to assess the prevalence of OD and its main risk factors and complications, as a tool to evaluate the effect of a specific therapy, and even as a reference test to validate a screening tool like the EAT-10 [40]. Although widely used to evaluate the prevalence of OD in different phenotypes of dysphagic patients (Table 5), very few studies considered the likelihood ratios (mainly LHR+) to assess the true (post-test) prevalence of OD [64].

It also has been recommended in several guidelines to manage patients with OD, and very often has been used as a clinical tool to diagnose OD, as it is not always possible to perform an instrumental exploration (FEES or VFS) of all patients at risk of OD, such as those in psychogeriatric units, special-needs schools, nursing homes, etc. [6] We believe that the V-VST must also be considered a clinical diagnostic tool for OD when instrumental evaluation is not available in a particular clinical setting, especially if the prevalence of OD is high, as discussed in the SR. This is because the V-VST can also provide information on the specific signs and mechanisms of impaired safety, aspirations, and efficacy of swallow in these patients, and the test is very useful to develop a therapeutic plan based on the identification of these main pathophysiological elements, as well as on the effect of different viscosity levels, to allow a safe swallow for each patient [6]. Its versatility, strong link with a therapeutic decision for fluid thickening, and good psychometric properties make this test one of those chosen most often by professionals from different countries as a clinical diagnostic tool. Other reviews that compared the V-VST with other bedside clinical tests confirmed that it is one of the best options to diagnose OD in adult patients with dysphagia if appropriate training is provided to the healthcare professionals [32].

Prevalence of dysphagia in older adults is very high: up to 50% of elderly patients admitted to acute hospitals, 70% of elderly patients in nursing homes, and 27% of elderly citizens still living in the community present swallowing dysfunctions leading to oropharyngeal dysphagia that results in malnutrition and dehydration [30,62]. This fact directs us to the need for a systematic diagnosis in our community. The V-VST helps clinicians not only to detect these patients, but also to provide an accurate prescription of the viscosity of the alimentary fluids and thickened fluids they need to maintain their nutritional and hydration status safely. The sensitivity of the V-VST in selecting the patients needing thickened fluids was 84.6% with a specificity of 73.4 [7], further suggesting its utility for nutritional professionals in the clinical setting.

In addition, the V-VST can be used in research. It has been already applied as an index test to assess patients and to evaluate the outcomes of swallowing therapies. We believe it can be a very useful assessment tool for food and nutrition scientists: first, to confirm the effectiveness of new pre-thickened products and the different fluid foods prescribed to the patients; and second, to monitor the progression and evolution of the patients and re-adjust the prescribed viscosity level according to the natural history of their diseases.

#### The V-VST

The V-VST was designed as an effort test to detect patients’ signs of impaired safety and efficacy of swallow through an algorithm that protects dysphagic patients from the risk of aspiration during the evaluation. It uses intermediate viscosity in the initial trial, and is the only test that can help with fluid thickening and compensatory management decision making [7]. Even during the COVID-19 pandemic, when instrumental evaluations have been suppressed in the majority of countries due to risk of infection of healthcare professionals, the V-VST has become a very useful tool to diagnose OD in this cohort of patients [10]. Our review also showed the heterogeneity in the viscosity descriptors and objective viscosity values used in the SR, but especially in the ScR. However, in order to be consistent, standardization regarding the use of descriptors and viscosities should be achieved. We noted that, between studies, a single descriptor such as “pudding” could refer to a wide range of bolus viscosities when measured in International System units (mPa-s) (i.e., a descriptor could be related to very different viscosities in mPa-s, depending on the author). Thus, viscosity should be reported in International System units (mPa-s) in order to standardize and globalize the use of thickened fluids for the clinical diagnosis of OD with volume-viscosity-based algorithms and for OD compensatory strategies. Recent studies with new xanthan-gum thickeners from our group clearly showed that the therapeutic range of these thickening agents is from 250 mPa-s to 800–1000 mPa-s, and therefore two viscosity levels of 250 mPa-s and 800 mPa-s for impaired safety and aspiration are recommended with these new products when using the V-VST in future studies [65].

### 4.3. Study Limitations

There were some limitations to our research; the main one was that the literature available on the validation of V-VST psychometrics is still limited. Even though further studies should be developed in the future, we were able to undertake an exhaustive review of the ones that finally were included according to the stated criteria. The aim of the study was achieved; however, not all studies published data on the same endpoints (some published the validity for OD; others for impaired safety or impaired efficacy), and for this reason, our meta-analysis was performed by combining different studies for each endpoint. It was not possible to conduct a meta-analysis that included all four studies for all endpoints. Thus, there is a need to further investigate the psychometric characteristics of the test, and also its reliability for other specific underlying pathologies. Finally, a tendency to only publish positive results could be a potential bias in the results of this systematic review. Another limitation of this study was that only one search engine (PubMed) was used in the ScR. Further studies adding more resources could broaden our findings.

## 5. Conclusions

The V-VST has been validated as a screening tool for OD and aspirations in few studies. It is used internationally by many researchers and clinicians. It is an easy, cost-effective, safe, and reliable tool to use not only as a screening test, but also as a clinical diagnostic tool when instrumental assessment is not available. It has a high discriminating ability for OD and the signs of impairments on swallowing function if performed by a trained healthcare professional. It also enables the examiner to provide therapeutic recommendations on fluid thickening for the safest and most effective swallow for the patient, or to refer them for a more comprehensive instrumental diagnosis evaluation when needed. This clinical tool has been validated in several phenotypes of dysphagic patients with a high Se and Sp, and this might lead to reducing the risk for nutritional and respiratory complications and direct them to a better and quicker management, improving their quality of life and health status, as well as reducing the high economic impact of OD. In summary, we recommend its universal use in the systematic screening and clinical diagnosis of OD [43,65,66].

## Figures and Tables

**Table 1:** Search terms used in the bibliographic search for PubMed. Our search strategy combined 3 arms that merged the terms with the Boolean ”or” inside each arm and the Boolean “and” between arms for the systematic review (SR). For the scoping review (ScR), we only applied the V-VST arm.

Dysphagia	Diagnostics	V-VST
Deglutition (MeSH)	sensitive * (ti/abs)	Volume viscosity Swallowing test
Deglutition disorders (MeSH)	sensitivity and specificity (MeSH)	V-VST
Deglutition disorders/diagnosis (MeSH)	diagnose (ti/abs)	volume-viscosity swallow test
Deglutition disorders/nursing (MeSH)	diagnosed (ti/abs)	
Swallow (ti/abs)	diagnoses (ti/abs)	
Dysphagia (tw)	diagnosing (ti/abs)	
Dysphag */	diagnosis (ti/abs)	
Dysphag * (ti/abs)	diagnostic (ti/abs)	
Deglut * (ti/abs)	diagnosis (MeSH:noexp)	
	diagnostic * (MeSH:noexp)	
	diagnosis, differential (MeSH:noexp)	
	diagnosis (Subheading:noexp)	

* Papers excluded from SR that were already included in the ScR through the initial search.

**Table 2:** Summary table of the psychometric properties of the volume-viscosity swallow test (V-VST) for oropharyngeal dysphagia (OD) and all the clinical signs of impaired safety and efficacy of swallow evaluated with the test reported in each of the included studies in the systematic review (SR). Each of the reported studies included information on the phenotype of patients, sample size of the studies, and reference standard used.

**Title**	Accuracy of the V-VST for clinical screening OD				Clinical screening of OD in patients with ALS				Usefulness of the V-VST for screening dysphagia in subacute stroke patients in rehabilitation income				Sensitivity and specificity of the EAT and the V-VST for clinical evaluation of OD				**Value Ranges**				**Mean**				
**Author**	P. Clavé et al.				G. Paris et al.				A. Guillén-Solà et al.				L. Rofes et al.												
**Year**	2008 (7)				2012 (8)				2013 (9)				2014 (6)												
**Patient Phenotype**	Elderly, NDD, H&N, HV				ALS				Subacute stroke patients				Ageing, stroke, NDD, HV												
**Sample Size**	97: 40/24/21/12				20				52				134												
**Reference Standard**	VFS				VFS				VFS				VFS												
**Psychometrics**	**Se**	**Sp**	**PPV**	**NPV**	**Se**	**Sp**	**PPV**	**NPV**	**Se**	**Sp**	**PPV**	**NPV**	**Se**	**Sp**	**PPV**	**NPV**	**Se**	**Sp**	**PPV**	**NPV**	**Se**	**Sp**	**PPV**	**NPV**	**n total**
**OD**					93	80	100	83.3					94.0	88.0	98.0	70.0	98.8–93	88–25	100–96.4	83.3–50	93.2 *	81.4 *	95.2	65.0	251
**Impaired Safety**	88.2	64.7	90.9	57.9					84.2	64.3	86.4	60	87.0	81.0	93.0	46.0	88.2–84.2	81–64.3	93.0–86.4	60–46.0	86.1 *	68.5 *	90.1	56.3	283
**Penetrations**	83.7	64.7	87.2	57.9					34.3	70.6	70.6	34.3					83.7–34.3	70.6–64.7	87.2–70.6	57.9–34.3	59.0	67.7	78.9	46.1	149
**Aspirations**	100	28.8	28.8	100					88.2	71.4	60.0	92.6	91.0	28.0	21.0	94.0	100–88.2	71.4–28.0	60–21	100–92.6	93.1	42.3	36.6	76.1	283
**Cough**									82.4	54.3	46.7	86.4					82.4	54.3	46.7	86.4	82.4	54.3	46.7	86.4	52
**Voice Change**									80.0	50.0	34.8	88.2					80.0	50.0	34.8	88.2	80.0	50.0	34.8	88.2	52
**O_2_ Desaturation (>3%)**									41.2	97.1	88	77.3					41.2	97.1	88	77.3	41.2	97.1	88	77.3	52
**Impaired Efficacy**	92.4	33.3	94.8	25.0									79.0	75.0	93.0	67.0	92.4–79	75-33.3	94.8–93	67-25	85.7	54.2	93.0	67.0	231
**Oral Residue**	69.2	80.6	39.1	93.5					93.7	65	81.1	86.6					93.7–69.2	80.6–65	81.1–39.1	93.5–86.6	81.5	72.8	60.1	90.1	149
**Pharyngeal Residue**	86.4	34.6	75.0	52.9					40.0	70.8	55.5	55.9					86.4–40	70.8–34.6	75–55.5	55.9–52.9	63.2	52.7	65.3	54.4	149
**Piecemeal Deglutition**	88.4	87.5	96.8	63.6													88.4	87.5	96.8	63.6	88.4	87.5	96.8	63.6	97

V-VST: volume-viscosity swallow test; OD: oropharyngeal dysphagia; NDD: neurodegenerative diseases; H&N: head and neck cancer; HV: healthy volunteers; ALS: amyotrophic lateral sclerosis; VFS: videofluoroscopy; Se: sensitivity; Sp: specificity; PPV: positive predictive value; NPV: negative predictive value; n: number of participants; O_2_: oxygen. * Calculated with fixed-effect model.

**Table 3:** Summary of the main psychometric properties of the volume-viscosity swallow test (V-VST) for oropharyngeal dysphagia (OD) and all the clinical signs of impaired safety and efficacy of swallow evaluated with the test.

	Sensitivity	Specificity	LHR+	LHR−	DOR
OD	93.2 *	81.4 *	5.01	0.08	51.18
Impaired Safety	86.1 *	68.5 *	2.73	0.20	11.67
Penetrations	59.0	67.7	1.82	0.61	4.32
Aspirations	93.1	42.3	1.63	0.16	10.17
Cough	82.4	54.3	1.80	0.32	5.56
Voice Change	80.0	50.0	1.60	0.40	4.00
O_2_ Desaturation (>3%)	41.2	97.1	14.21	0.61	23.46
Impaired Efficacy	85.7	54.2	1.87	0.26	14.07
Oral Residue	81.5	72.8	2.99	0.25	11.89
Pharyngeal Residue	63.2	52.7	1.34	0.70	2.51
Piecemeal Deglutition	88.4	87.5	7.07	0.13	53.34

OD: oropharyngeal dysphagia; LHR: likelihood ratio; DOR: diagnostic odds ratio. * Calculated with fixed-effect model.

**Table 4:** Authors, years, countries, and quality of the publications included in the systematic (SR) and scoping (ScR) reviews. Bold depicts studies included in the SR, and no bold indicates studies included in the ScR.

Author	Year	Country	Title	Quality *
Clavé et al. [7]	2008	Spain	Accuracy of the volume-viscosity swallow test for clinical screening of oropharyngeal dysphagia and aspiration.	Sufficient ^1^; Sufficient (8/9) ^2^; High Quality ^3^
Gómez-Busto et al. [28]	2009	Spain	Approach to dysphagia in advanced dementia.	
Silveira Guijarro et al. [29]	2011	Spain	Oropharyngeal dysphagia in elderly inpatients in a unit of convalescence.	
Paris et al. [8]	2012	France	Clinical screening of oropharyngeal dysphagia in patients with ALS.	Sufficient ^1^; High Quality ^3^
Rofes et al. [3]		Spain	The volume-viscosity swallow test for clinical screening of dysphagia and aspiration.	Sufficient (8/9) ^2^
Serra-Prat et al. [30]		Spain	Oropharyngeal dysphagia as a risk factor for malnutrition and lower respiratory tract infection in independently living older persons: a population-based prospective study.	0 (low risk) ^4^
Guillén-Solà et al. [9]	2013	Spain	Usefulness of the volume-viscosity swallow test for screening dysphagia in subacute stroke patients in rehabilitation income.	Sufficient ^1^; High Quality ^3^
Almirall et al. [31]		Spain	Oropharyngeal dysphagia is a risk factor for community-acquired pneumonia in the elderly.	2 (low risk) ^4^
Kertscher et al. [32]		Netherlands	Bedside screening to detect oropharyngeal dysphagia in patients with neurological disorders: an updated systematic review.	Sufficient ^1^
Rofes et al. [6]	2014	Spain	Sensitivity and specificity of the eating assessment tool and the volume-viscosity swallow test for clinical evaluation of oropharyngeal dysphagia.	Sufficient ^1^; High Quality ^3^
Carrión et al. [5]	2015	Spain	Oropharyngeal dysphagia is a prevalent risk factor for malnutrition in a cohort of older patients admitted with an acute disease to a general hospital.	1 (low risk) ^4^
Miarons et al. [33]	2016	Spain	Drugs related to oropharyngeal dysphagia in older people.	1 (low risk) ^4^
Vilardell et al. [34]		Spain	A comparative study between modified starch and xanthan gum thickeners in post-stroke oropharyngeal dysphagia.	
Jørgensen et al. [26]	2017	Denmark	Interrater reliability of the volume-viscosity swallow test; screening for dysphagia among hospitalized elderly medical patients.	
Vilardell et al. [35]		Spain	Videofluoroscopic assessment of the pathophysiology of chronic poststroke oropharyngeal dysphagia.	
Melgaard et al. [36]		Denmark	The prevalence of oropharyngeal dysphagia in Danish patients hospitalised with community-acquired pneumonia.	1 (low risk) ^4^
Vilardell et al. [37]		Spain	Cough reflex attenuation and swallowing dysfunction in sub-acute post-stroke patients: prevalence, risk factors, and clinical outcome.	2 (low risk) ^4^
Mamolar et al. [38]		Spain	Swallowing disorders in Parkinson’s disease.	2 (low risk) ^4^
Melgaard et al. [39]	2018	Denmark	The prevalence of oropharyngeal dysphagia in acute geriatric patients.	0 (low risk) ^4^
Fernández-Rosati et al. [40]		Chile	Validation of the EAT-10 score to detect dysphagia in older people.	
Ye et al. [41]		China	Comparison of two bedside evaluation methods of dysphagia in patients with acute stroke.	
Miarons et al. [42]		Spain	Increased levels of substance P in patients taking beta-blockers are linked with a protective effect on oropharyngeal dysphagia.	0 (low risk) ^4^
Westmark et al. [43]		Denmark	The cost of dysphagia in geriatric patients.	
Wegner et al. [44]		Brazil	Oropharyngeal deglutition, nutrition, and quality of life in individuals with chronic pulmonary disease.	3 (low risk) ^4^
Michel et al. [45]		France	Oropharyngeal dysphagia in community-dwelling older patients with dementia: prevalence and relationship with geriatric parameters.	1 (low risk) ^4^
Rofes et al. [46]		Spain	Prevalence, risk factors and complications of oropharyngeal dysphagia in stroke patients: a cohort study.	0 (low risk) ^4^
Zamora Mur et al. [47]		Spain	Importance of the detection of dysphagia in geriatric patients.	2 (low risk) ^4^
Spronk et al. [48]	2019	Netherlands	Prevalence and characterization of dysphagia in hospitalized patients.	1 (low risk) ^4^
Peñalva-Arigita et al. [49]		Spain	Prevalence of dysphagia in a regional hospital setting: acute care hospital and a geriatric sociosanitary care hospital: a cross-sectional study.	2 (low risk) ^4^
Fernández-Pombo et al. [50]		Spain	Lesion location and other predictive factors of dysphagia and its complications in acute stroke.	0 (low risk) ^4^
Arreola et al. [51]		Spain	Natural history of swallow function during the three-month period after stroke.	1 (low risk) ^4^
Wang et al. [52]		China	Effects of capsaicin on swallowing function in stroke patients with dysphagia: a randomized controlled trial.	
Mayer Silva da Cunha et al. [53]	2020	Brasil	Symptoms suggestive of dysphagia and the quality of life in cocaine and/or crack users.	
Benfield et al. [27]		UK	Accuracy and clinical utility of comprehensive dysphagia screening assessments in acute stroke: a systematic review and meta-analysis.	
Liu et al. [54]		China	Impact of the systematic use of volume-viscosity swallow test in patients with acute ischaemic stroke: a retrospective study.	
Mateos-Nozal et al. [55]		Spain	High prevalence of oropharyngeal dysphagia in acutely hospitalized patients aged 80 and older.	
Melgaard et al. [56]		Denmark	Systematic dysphagia screening of elderly persons in the emergency department—a feasibility study.	3 (low risk) ^4^

* Quality evaluated by: ^1^ Criteria for methodological quality assessment of studies (Dutch Cochrane Centre) [19]; ^2^ Nine-item methodological assessment of quality from Bours et al. [57]; ^3^ GRADE (Grading of Recommendations Assessment, Development, and Evaluation) [21]; and ^4^ Quality assessment checklist for prevalence studies (adapted from Hoy et al.) [58]. 0–3 indicates low risk.

**Table 5:** Prevalence of oropharyngeal dysphagia (OD) in different phenotypes of patients and target populations according to the studies included in the scoping review using the volume-viscosity swallow test as a tool to clinically diagnose OD.

Phenotype	Target Population	Prevalence %	References
**Older**	Community	86.6	Michel (2018) [45]
		40.3	Almirall (2013) [62]
		25.0	Serra-Prat (2012) [30]
	Hospitalized AGU	30.7	Spronk (2019) [48]
		28.5	Peñalva-Arigita (2019) [49]
		50.0	Melgaard (2018) [39]
		50.0	Miarons (2018) [42]
		86.0	Zamora Mur (2018) [47]
		41.9	Miarons (2016) [33]
		47.4	Carrión (2015) [5]
		82.4	Mateos-Nozal (2020) [55]
		28.42	Melgaard (2020) [56]
	Hospitalized with CAP	34.4	Melgaard (2017) [36]
		91.7	Almirall (2013) [62]
	Rehabilitation centre with COPD	52.9 Impaired efficacy 11.8 Impaired efficacy and safety	Wegner (2018) [44]
**NDD**	Parkinson	78.9	Mamolar (2017) [38]
Stroke	Acute phase	56.6	Fernandez-Pombo (2019) [50]
		39.7	Arreola (2019) [51]
		45.1	Rofes (2018) [46]
	Hospitalized AGU Chronic phase Acute phase	54.7	Liu et al. (2020) [54]
		41.7	Arreola (2019) [51]
		60.4 Impaired safety 95.9 Impaired efficacy	Vilardell (2017) [35]

NDD: neurodegenerative disease; AGU: acute geriatric unit; CAP: community-acquired pneumonia; COPD: chronic obstructive pulmonary disease.

## Data Availability

The data presented in this study are available in this article and Supplementary Materials here.

## Supplementary Materials

The following are available online at https://www.mdpi.com/article/10.3390/foods10081900/s1, Brief description of the rheological characterization of bolus viscosity in the V-VST of the included studies. Table S1: Summary of the conclusions of each study included in the integrative review. Table S2: Thickeners, viscosity descriptor levels, viscosity values (IS units), and grams of thickening agent/100 mL water used in the publications included in the SR and ScR. Figure S1: Nomogram for oropharyngeal dysphagia (OD), impaired safety (IS), impaired efficacy (IE), and aspirations combining the resultant LHR+ (in blue) and LHR– (in red) for a pre-test prevalence of 10%, 50%, and 75%.

## References

[1] International Statistical Classification of Diseases and Related Health Problems. http://apps.who.int/classifications/apps/icd/%0Aicd10online/?gr10.htm±r13.

[2] Clavé P., Shaker R. (2015). Dysphagia: Current reality and scope of the problem. Nat. Rev. Gastroenterol. Hepatol..

[3] Rofes L., Arreola V., Clave P. (2012). The Volume-Viscosity Swallow Test for Clinical Screening of Dysphagia and Aspiration. Issues Complement. Feed..

[4] Carrión S., Roca M., Costa A., Arreola V., Ortega O., Palomera E., Serra-Prat M., Cabré M., Clavé P. (2017). Nutritional status of older patients with oropharyngeal dysphagia in a chronic versus an acute clinical situation. Clin. Nutr..

[5] Carrión S., Cabré M., Monteis R., Roca M., Palomera E., Serra-Prat M., Rofes L., Clavé P. (2015). Oropharyngeal dysphagia is a prevalent risk factor for malnutrition in a cohort of older patients admitted with an acute disease to a general hospital. Clin. Nutr..

[6] Rofes L., Arreola V., Mukherjee R., Clavé P. (2014). Sensitivity and specificity of the Eating Assessment Tool and the Volume-Viscosity Swallow Test for clinical evaluation of oropharyngeal dysphagia. Neurogastroenterol. Motil..

[7] Clavé P., Arreola V., Romea M., Medina L., Palomera E., Serra-Prat M. (2008). Accuracy of the volume-viscosity swallow test for clinical screening of oropharyngeal dysphagia and aspiration. Clin. Nutr..

[8] Paris G., Martinaud O., Hannequin D., Petit A., Cuvelier A., Guedon E., Ropenneck P., Verin E. (2012). Clinical screening of oropharyngeal dysphagia in patients with ALS. Ann. Phys. Rehab. Med..

[9] Guillén-Solà A., Marco E., Martínez-Orfila J., Mejías M.F.D., Passalacqua M.D., Duarte E., Escalada F. (2013). Usefulness of the volume-viscosity swallow test for screening dysphagia in subacute stroke patients in rehabilitation income. Neurorehabilitation.

[10] Biomedical Research Center in Network of Liver and Digestive Diseases [CIBERehd] SC de D [SCD] and H de M [HM] (2020). Basic Procedures to Assess and Treat Oropharyngeal Dysphagia in Patients with Covid-19 Infection. Expert Opinion Practical Guidance from Hospital De Mataró, Catalonia, Spain. https://www.rcslt.org/-/media/docs/Covid/RCSLT-PPE-guidance.

[11] Espinosa-Val M.C., Martín-Martínez A., Graupera M., Arias O., Elvira A., Cabré M., Palomera E., Bolívar-Prados M., Clavé P., Ortega O. (2020). Prevalence, Risk Factors, and Complications of Oropharyngeal Dysphagia in Older Patients with Dementia. Nutrients.

[12] Cooper S., Cant R., Kelly M., Levett-Jones T., McKenna L., Seaton P., Ng L., Borgossian F. (2019). Online Supplemental Material. Table 3. The Scoping Review Checklist. Clin. Nurs. Res..

[13] Riera S., Marin S., Serra M., Clave P. A Systematic Review to Assess the Psychometrics of the Volume Viscosity Swallow Test (V-VST) on the Clinical Diagnosis of Oropharyngeal Dysphagia. https://www.crd.york.ac.uk/prospero/display_record.php?RecordID=136252.

[14] Campbell J., Kulgar M., Ding S., Carmody D., Hakonsen S., Jadotte Y., Ws C. (2020). Diagnostic Test Accuracy Systematic Reviews. JBI Manual for Evidence Synthesis.

[15] Liberati A., Altman D.G., Tetzlaff J., Mulrow C.D., Gøtzsche P.C., Ioannidis J.P.A., Clarke M., Devereaux P., Kleijnen J., Moher D. (2009). The PRISMA Statement for Reporting Systematic Reviews and Meta-Analyses of Studies That Evaluate Health Care Interventions: Explanation and Elaboration. PLoS Med..

[16] Deeks J.J. (1994). Meta-Analysis, Decision Analysis, and Cost-Effectiveness Analysis. Methods for Quantitative Synthesis in Medicine.

[17] CEBM The Centre for Evidence-Based Medicine Develops, Promotes and Disseminates Better Evidence for Healthcare. https://www.cebm.net/2014/02/likelihood-ratios/.

[18] Caraguel C.G.B., Vanderstichel R. (2013). The two-step Fagan’s nomogram: Ad hoc interpretation of a diagnostic test result without calculation. Evid. Based Med..

[19] Cochrane Collaboration (2011). Cochrane Handbook for Diagnostics Test Accuracy. http://srdta.cochrane.org/handbook-dta-reviews.

[20] Whiting P.F., Rutjes A.W., Westwood M.E., Mallett S., Deeks J.J., Reitsma J.B., Leeflang M.M., Sterne J.A., Bossuyt P.M. (2011). The QUADAS-2 Group: QUADAS-2: A revised tool for the quality assessment of diagnostic accuracy studies. Ann. Intern. Med..

[21] Guyatt G., Oxman A.D., Akl E.A., Kunz R., Vist G., Brozek J., Norris S., Falck-Ytter Y., Glasziou P., Debeer H. (2011). GRADE guidelines: Introduction—GRADE evidence profiles and summary of findings tables. J. Clin. Epidemiol..

[22] Cooper S., Cant R., Kelly M., Levett-Jones T., McKenna L., Seaton P., Bogossian F. (2021). An Evidence-Based Checklist for Improving Scoping Review Quality. Clin. Nurs. Res..

[23] Equator (2009). STROBE_checklist_v4_combined. https://www.equator-network.org/reporting-guidelines/strobe/.

[24] Cuschieri S. (2019). The STROBE guidelines. Saudi J. Anaesth..

[25] Hoy D., Brooks P., Woolf A., Blyth F., March L., Bain C., Baker P., Smith E., Buchbinder R. (2012). Assessing risk of bias in prevalence studies: Modification of an existing tool and evidence of interrater agreement. J. Clin. Epidemiol..

[26] Jørgensen L.W., Søndergaard K., Melgaard D., Warming S. (2017). Interrater reliability of the Volume-Viscosity Swallow Test; screening for dysphagia among hospitalized elderly medical patients. Clin. Nutr. ESPEN.

[27] Benfield J.K., Everton L.F., Bath DSc F.P.M., England T.J. (2020). Accuracy and clinical utility of comprehensive dysphagia screening assessments in acute stroke: A systematic review and meta-analysis. J. Clin. Nurs..

[28] Gómez-Busto F., Andia V., De Alegria L.R., Francés I. (2009). Abordaje de la disfagia en la demencia avanzada. Rev. Esp. Geriatr. Gerontol..

[29] Silveira Guijarro L.J., Domingo García V.D., Montero Fernández N., Osuna del Pozo C.M., Álvarez Nebreda L., Serra-Rexach J.A. (2011). Disfagia orofaríngea en ancianos ingresados en una unidad de convalecencia. Nutr. Hosp..

[30] Serra-Prat M., Palomera M., Gómez C., Sar-Shalom D., Saiz A., Montoya J.G., Navajs M., Palomera E., Clave P. (2012). Oropharyngeal dysphagia as a risk factorfor malnutrition and lower respiratory tractinfection in independently living olderpersons: A population-based prospective study. Age Ageing.

[31] Almirall J., Cabré M., Clave P. (2013). Complications of Oropharyngeal Dysphagia: Aspiration Pneumonia. Issues Complement. Feed..

[32] Kertscher B., Speyer R., Palmieri M., Plant C. (2013). Bedside Screening to Detect Oropharyngeal Dysphagia in Patients with Neurological Disorders: An Updated Systematic Review. Dysphagia.

[33] Miarons M., Campins L., Palomera E., Serra-Prat M., Cabré M., Rofes L. (2016). Drugs Related to Oropharyngeal Dysphagia in Older People. Dysphagia.

[34] Vilardell N., Rofes L., Arreola V., Speyer R., Clavé P. (2016). A Comparative Study between Modified Starch and Xanthan Gum Thickeners in Post-Stroke Oropharyngeal Dysphagia. Dysphagia.

[35] Vilardell N., Rofes L., Arreola V., Martin A., Muriana D., Palomeras E., Ortega O., Clavé P. (2017). Videofluoroscopic assessment of the pathophysiology of chronic poststroke oropharyngeal dysphagia. Neurogastroenterol. Motil..

[36] Melgaard D., Baandrup U., Bøgsted M., Bendtsen M.D., Hansen T. (2017). The Prevalence of Oropharyngeal Dysphagia in Danish Patients Hospitalised with Community-Acquired Pneumonia. Dysphagia.

[37] Vilardell N., Rofes L., Nascimento W.V., Muriana D., Palomeras E., Clavé P. (2017). Cough reflex attenuation and swallowing dysfunction in sub-acute post-stroke patients: Prevalence, risk factors, and clinical outcome. Neurogastroenterol. Motil..

[38] Mamolar Andrés S., Santamaria Rabanal M.L., Granda Membiela C.M., Fernández Gutiérrez M.J., Sirgo Rodríguez P., Álvarez Marcos C. (2017). Trastornos de la deglución en la enfermedad de Parkinson. Acta Otorrinolaringol. Esp..

[39] Melgaard D., Rodrigo-Domingo M., Mørch M.M. (2018). The Prevalence of Oropharyngeal Dysphagia in Acute Geriatric Patients. Geriatrics.

[40] Fernández-Rosati J., Lera L., Fuentes-López E., Albala C. (2018). Validation of the eat-10 score to detect dysphagia in older people. Rev. Med. Chile.

[41] Ye T., Huang S., Dong Y., Dong Q. (2018). Comparison of two bedside evaluation methods of dysphagia in patients with acute stroke. Stroke Vasc. Neurol..

[42] Miarons M., Tomsen N., Nascimento W., López-Faixó D., Clavé P., Rofes L. (2018). Increased levels of substance P in patients taking beta-blockers are linked with a protective effect on oropharyngeal dysphagia. Neurogastroenterol. Motil..

[43] Westmark S., Melgaard D., Rethmeier L.O., Ehlers L.H. (2018). The cost of dysphagia in geriatric patients. Clin. Outcomes Res..

[44] Wegner D.A., Steidl E.M.S., Pasqualoto A.S., Mancopes R. (2018). Oropharyngeal deglutition, nutrition, and quality of life in individuals with chronic pulmonary disease. Codas.

[45] Michel A., Vérin E., Gbaguidi X., Druesne L., Roca F., Chassagne P. (2018). Oropharyngeal Dysphagia in Community-Dwelling Older Patients with Dementia: Prevalence and Relationship with Geriatric Parameters. J. Am. Med. Dir. Assoc..

[46] Rofes L., Muriana D., Palomeras E., Vilardell N., Alvarez-Berdugo D., Casado V., Clavé P. (2018). Prevalence, risk factors and complications of oropharyngeal dysphagia in stroke patients: A cohort study. Neurogastroenterol. Motil..

[47] Zamora M.A., Palacín A.C., Guardia C.A.I., Zamora C.A., Clemente R.E., Santaliestra G.J. (2018). Importance of the detection of dysphagia in geriatric patients. Semergen.

[48] Spronk P.E., Spronk L.E.J., Lut J., Gnacke E., Mijnes D., Van Munster B., Kröner A. (2019). Prevalence and characterization of dysphagia in hospitalized patients. Neurogastroenterol. Motil..

[49] Peñalva-Arigita A., Prats R., Lecha M., Sansano A., Vila L. (2019). Prevalence of dysphagia in a regional hospital setting: Acute care hospital and a geriatric sociosanitary care hospital: A cross-sectional study. Clin. Nutr. ESPEN.

[50] Fernández-Pombo A., Seijo-Raposo I.M., López-Osorio N., Cantón-Blanco A., González-Rodríguez M., Arias-Rivas S., Rodríguez-Yáñez M., Santamaría-Nieto A., Díaz-Ortega C., Gómez-Vázquez E. (2019). Lesion location and other predictive factors of dysphagia and its complications in acute stroke. Clin. Nutr. ESPEN.

[51] Arreola V., Vilardell N., Ortega O., Rofes L., Muriana D., Palomeras E., Álvarez-Berdugo D., Clavé P. (2019). Natural History of Swallow Function during the Three-Month Period after Stroke. Geriatrics.

[52] Wang Z., Wu L., Fang Q., Shen M., Zhang L., Liu X. (2019). Effects of capsaicin on swallowing function in stroke patients with dysphagia: A randomized controlled trial. J. Stroke Cerebrovasc. Dis..

[53] Mayer Silva da Cunha K., de Campos Moreira T., Tamanini de Almeida S., Tannhauser Barros H.M., Ferigolo M. (2020). Symptoms Suggestive of Dysphagia and the Quality of Life in Cocaine and/or Crack Users. Dysphagia.

[54] Liu Z.-Y., Zhang X.-P., Mo M.-M., Ye R.-C., Hu C.-X., Jiang M.-Q., Lin M.-Q. (2020). Impact of the systematic use of the volume-viscosity swallow test in patients with acute ischaemic stroke: A retrospective study. BMJ Open.

[55] Mateos-Nozal J., Montero-Errasquín B., García E.S., Rodríguez E.R., Cruz-Jentoft A.J. (2020). High Prevalence of Oropharyngeal Dysphagia in Acutely Hospitalized Patients Aged 80 Years and Older. J. Am. Med. Dir. Assoc..

[56] Melgaard D., Sørensen L.R., Lund D., Leutscher P., Ludwig M. (2020). Systematic Dysphagia Screening of Elderly Persons in the Emergency Department—A Feasibility Study. Geriatrics.

[57] Bours G.J.J.W., Speyer R., Lemmens J., Limburg M., De Wit R. (2009). Bedside screening tests vs. videofluoroscopy or fibreoptic endoscopic evaluation of swallowing to detect dysphagia in patients with neurological disorders: Systematic review. J. Adv. Nurs..

[58] Hoy D., Brooks P., Woolf A., Blyth F., March L., Bain C. (2012). S2 Table. Quality assessment checklist for prevalence studies. J. Clin. Epidemiol..

[59] Altman D., Bland J., Massif E.B. (1994). Diagnostic tests. 1: Sensitivity and specificity. BMJ.

[60] McHugh M.L. (2012). Lessons in biostatistics interrater reliability: The kappa statistic. Biochem. Med..

[61] Baijens L.W., Clavé P., Cras P., Ekberg O., Forster A., Kolb G.F., Leners J.C., Masiero S., del Nozal J.M., Ortega O. (2016). European Society for Swallowing Disorders—European Union Geriatric Medicine Society white paper: Oropharyngeal dysphagia as a geriatric syndrome. Clin. Interv. Aging.

[62] Almirall J., Rofes L., Serra-Prat M., Icart R., Palomera E., Arreola V., Clavé P. (2012). Oropharyngeal dysphagia is a risk factor for community-acquired pneumonia in the elderly. Eur. Respir. J..

[63] Martino R., Silver F., Teasell R., Bayley M., Nicholson G., Streiner D.L., Diamant N.E. (2009). The toronto bedside swallowing screening test (TOR-BSST) development and validation of a dysphagia screening tool for patients with stroke. Stroke.

[64] Serra-Prat M., Hinojosa G., López D., Juan M., Fabré E., Voss D.S., Calvo M., Marta V., Ribó L., Palomera E. (2011). Prevalence of oropharyngeal dysphagia and impaired safety and efficacy of swallow in independently living older persons. J. Am. Geriatr. Soc..

[65] Bolivar-Prados M., Rofes L., Arreola V., Guida S., Nascimento W.V., Martin A., Vilardell N., Fernández O.O., Ripken D., Lansink M. (2019). Effect of a gum-based thickener on the safety of swallowing in patients with poststroke oropharyngeal dysphagia. Neurogastroenterol. Motil..

[66] Marin S., Serra-Prat M., Ortega O., Clavé P. (2018). Cost of oropharyngeal dysphagia after stroke: Protocol for a systematic review. BMJ Open.

