# Supplementary material for: A Systematic and a Scoping Review on the Psychometrics and Clinical Utility of the Volume-Viscosity Swallow Test (V-VST) in the Clinical Screening and Assessment of Oropharyngeal Dysphagia
Source: Foods. 2021 Aug 16;10(8):1900. doi: 10.3390/foods10081900 (PMC8391460; doi:10.3390/foods10081900)
Supplement: Supplementary file 1 [file foods-10-01900-s001.zip › SUPPLEMENTARY MATERIAL.pdf]

SUPPLEMENTARY MATERIAL

Supplementary methods

Rheological Characterization of Bolus Viscosity in V-VSTof the inlcuded studies

We used a rotational viscometer (Thermo Fisher Scientific, Haake Viscotester®550, Germany) to analyze shear viscosity at 25ºC, in a shear rate range from 1 to 1,000 s^{-1}. Viscosity values were measured at the shear rate of 50 s^{-1}. An NV-rotor was used to analyze lower viscosities (<350mPa·s) and an SV-DIN rotor to analyze higher viscosities (>350mPa·s). RheoWin software, version 4.61 (Thermo Fisher Scientific, USA), was used for data processing. To prepare the thickened fluids for the analysis we followed the instructions described in the publications used for our paper as described in Supplementary table II.

Supplementary results

Table S1. Summary of the conclusions of each study included in the integrative review.

STUDY	CONCLUSIONS
	**PREVALENCES**
Silveira Guijarro (2011)	OD is highly prevalent among elderly patients. Only half of the cases are diagnosed through the specific anamnesis. The test detected a high prevalence of dysphagia so that its routine use is recommended specially in patients at risk taking into account the peculiarities of using it in the elderly.
Serra-Prat (2012)	OD is a risk factor for malnutrition and LRTI in independently living older subjects. Results suggest that older persons should be routinely screened and treated for OD to avoid nutritional and respiratory complications.
Almirall (2013)	In elderly subjects, oropharyngeal dysphagia is strongly associated with CAP, independently of functionality and comorbidities. Elderly patients with pneumonia presented a severe impairment of swallow and airway protection mechanisms. We recommend universal screening of dysphagia in older persons with pneumonia.
Carrión (2015)	Prevalence of dysphagia was higher than malnutrition in our older patients. Dysphagia was an independent risk factor for malnutrition, and both conditions were related to poor outcome.
Miarons (2016)	The present study is the first one to widely investigate the association between drugs and OD, increasing understanding of their association. The role of beta blockers in OD needs to be further studied as their potentially beneficial effects on the swallowing function in older patients could help to prevent complications.
Melgaard (2017)	OD is related to frailty and poor outcome.
Vilardell (2017)	Prevalence of subacute post-stroke OD and swallow safety impairments was much higher than CRT attenuation, and risk factors strongly differed suggesting that the swallow response receives a stronger cortical control than the cough reflex. OD has a greater impact on PSP clinical outcome than impaired cough, the poorest prognosis being for patients with both airway protective dysfunctions.
Mamolar (2017)	The SDQ test detected swallowing disorders in 36.5% of the subjects with Parkinson's disease. Disorders in swallowing efficiency and safety were demonstrated in 94.7% of this subset. Disorders of efficiency were more frequent than those of safety, establishing a relationship with greater time in ingestion and the appearance of respiratory pathology and pneumonias.
Melgaard (2018)	OD is prevalent in acute geriatric patients, and the mortality is 34% within six months of hospitalization. Screening for OD should be given more attention and included in geriatric guidelines.
Miarons (2018)	Patients TBB have higher serum and saliva SP levels in comparison with patients NTBB. They also found that patients TBB presented a lower prevalence of OD than patients NTBB.
Wegner (2018)	Changes in efficacy and safety of deglutition were observed; however, no nutritional risk was evidenced in the sample evaluated. Correlation between nutritional risk and quality of life in deglutition was also observed.
Michel (2018)	OD is very frequent in community-dwelling older persons with dementia and is associated with dependency and frailty. The V-VST is an easy-to-perform and well tolerated screening test in this population and therefore should be systematically included in the geriatric assessment of older persons with dementia. The role of V-VST in therapeutic strategies of OD remains to be evaluated.
Rofes (2018)	Post-stroke OD is prevalent and associated with poor short and long term prognosis. Stroke severity and patient status before stroke were more relevant to OD than lesion location. Systematic screening programs and early OD management could significantly improve post-stroke patient outcome.
Zamora Mur (2018)	Dysphagia has a significant mortality, and the use of thickeners after its detection should be properly reported.
Fernandez-Pombo (2019)	Post-stroke dysphagia was associated with the occurrence of respiratory tract infection and mortality. This study also provides more information about how certain demographic and clinical factors, as well as neuroimaging patterns, influence dysphagia. This fact may help to identify at an early stage those patients with a greater risk of developing swallowing alterations.
Arreola (2019)	It is important to assess swallowing function not only in the acute phase, but also in the chronic post-stroke phase in order to detect changes in swallowing and to establish the appropriate interventions to prevent poor outcome. In addition, as treatment of PS-OD is changing from compensatory strategies to the enhancement of brain plasticity, both to recover swallow function and to improve brain-

	related swallowing dysfunction, from their results, they suggest that randomized control studies assessing these new techniques should include control groups monitoring the spontaneous development of swallowing function.
Spronk (2019)	Training should be conducted for nursing staff to improve.
Peñalva- Argita (2019)	Prevalence of OD matches with what is described in the literature. There is a high proportion of infradiagnostic and overall inadequate treatment, more than a third of patients do not have their diet adapted correctly. It is important to identify this symptom to treat it properly and to raise awareness among sanitary professionals for best adaptation of treatment.
Mayer Silva da Cunha (2020)	The presence of symptoms suggestive of dysphagia was reported by a significant number of cocaine and/or crack users interviewed in this study. The main symptoms reported were the feeling of "food stuck in the throat". In the subjects submitted to the V-VST exam, the index of disturbance was low, mainly when compared to the results of the self-perception of the individuals through the EAT-10. The absence of solid consistency in the chosen test may have failed to identify patients who had swallowing difficulties only for this consistency. The complaints of sensation of "food trapped in the throat" and the need to make an effort to swallow solid foods reported by significant numbers of users contribute to this justification. The abstinence period of the substances used may also have interfered with this result, since the resolution of the symptoms suggestive of dysphagia can be given after a few days of cessation of cocaine and/ or crack use.
Mateos-Nozal (2020)	The prevalence of oropharyngeal dysphagia is high in the oldest old patients admitted to an acute geriatric unit when assessed with an objective diagnostic method (V-VST). Our findings suggest that objective swallowing assessment should be routinely performed on admission in order to start early interventions to avoid complications of dysphagia in this complex population.
Liu (2020)	The V-VST was a better clinical screening tool, and it can also provide detailed suggestions regarding dietary modifications to prevent aspiration and SAP.
	**REVIEWS AND GUIDELINES**
Gómez-Busto (2009)	V-VST is an easy test that can be performed in any assistential resource. Identifies properly those patients with safety swallow impairments that need to be restricted for liquids intake, and brings information of the most suitable bolus (volume and viscosity).
Rofes (2012)	V-VST is a sensitive tool to detect patients with OD and guide to dietary modifications when the VFS cannot be performed. It was designed to protect the patient of aspiration during the examination, and can be administered in 5 to 10 min by any member of the multidisciplinary dysphagia team.
Kertscher (2013)	V-VST and the TOR-BSST are the relevant bedside screening identified in this review. V-VST is the only tool that results in direct advice on the most suitable oral intake considering a patient's condition.
Ye (2018)	V-VST provided information on the severity of dysphagia, and also the strategy of management. V-VST may be more useful since it help in guiding the management of dysphagia in acute stroke patients.
Benfield (2020)	V-VST was one of the 3 included studies that underwent validity. It has been reported with the highest sensitivity and specificity for identifying dysphagia. it is designed to be carried out by non-specialists in dysphagia trained for the test and is reported to take just 5-10min to administer. Also, allows recommendations of diet, texture and volumes.
	**THERAPEUTIC EFFECT AND MISCELLANEOUS**
Vilardell (2015)	Authors studied 122 patients (46 starch, 76 xanthan gum). (A) V-VST showed that both thickeners similarly improved safety of swallow. The main result of this comparative study is that thickening liquids with both agents (MS and XG) present a strong therapeutic effect on safety of swallow in chronic post- stroke OD patients.
Fernandez-Rosati (2018)	The EAT-10 questionnaire is valid and reliable compared with the V-VST and can be used as a clinical instrument in primary care in our country to identify older people with dysphagia.
Miarons (2018)	Patients TBB have higher serum and saliva SP levels in comparison with patients NTBB. They also found that patients TBB presented a lower prevalence of OD than patients NTBB.
Westmark (2018)	They determined the cost at the hospital and in the municipality for geriatric patients with dysphagia to be significantly higher than for geriatric patients who did not have dysphagia.
Wang (2019)	Supplementation of natural capsaicin in thermal tactile stimulation and additional nectar viscosity boluses improves swallowing function in stroke patients with dysphagia. In light of these findings, it is conceivable that regular use of natural capsaicin might become a strategy to treat dysphagia in stroke.

Table S2. Thickeners, viscosity descriptor levels, viscosity values (SI units) and grams of thickening agent/100 mL water used in the publications included in the SR and ScR.

	Resource ThickenUp / Food thickener*			
**Descriptors**	**Grams/100ml water**	**Viscosities (mPa·s) published**	**Viscosities (mPa·s) calculated**	**References**
Nectar<br>Pudding or Spoon-thick	4.5<br>9	ND	98.61<br>4539.5	*Clavé (2008)*
Nectar<br>Pudding or Spoon-thick	4.5<br>9	270<br>3900	98.61<br>4539.5	*Serra-Prat (2012)*[2]
Nectar<br>Pudding or Spoon-thick	4.5<br>9	ND	98.61<br>4539.5	*Guillén-Solà (2013); Carrión (2015)*[2]*; Miarons (2016)*[2]*; Zamora-Mur (2018)*[2]*; Michel (2018)*[2]
Nectar<br>Pudding or Spoon-thick	4.5<br>9	ND	ND	*Paris (2012)*; Mamolar (2016)**
Nectar<br>Pudding or Spoon-thick	1.2<br>6	295.02<br>3682.21	ND	*Melgaard (2017,2018)* [5]
Nectar<br>Pudding or Spoon-thick	ND	ND	ND	*Vilardell (2017)*[2,5]*; Miarons (2018); Rofes (2018)*[4,5]*; Fernández-Pombo (2019); Peñalva-Argita(2019)*[1]
Nectar<br>Pudding or Spoon-thick	ND	250<br>3500	ND	*Arreola (2020)*

	Resource ThickenUp Clear			
**Descriptors**	**Grams/100ml water**	**Viscosities (mPa·s) published**	**Viscosities (mPa·s) calculated**	**References**
Nectar	1.2	238	83.47	*Rofes (2014)*
Honey	2.4	766	255.59	*Rofes (2014)*
Conservative spoon-thick	3.6	1098	377.11	*Rofes (2014)*
Extreme spoon-thick	6	1840	684.81	*Rofes (2014)*
Nectar<br>Extreme spoon-thick	1.2<br>6	ND	83.47<br>684.81	*Jorgensen (2017)*
Nectar<br>Extreme spoon-thick	1.2<br>6	ND	ND	*Spronk (2019)*

In: Almirall (2013), and Wegner (2018), there was no determination of the grammage or the thickener used to perform the V-VST. ND: not determined. Reference followed when applying the V-VST: [1] *Clavé (2004);* [2] *Clavé (2008); Rofes (2012);* [4] *Guillén-Solà (2013);* [5] *Rofes (2014).*

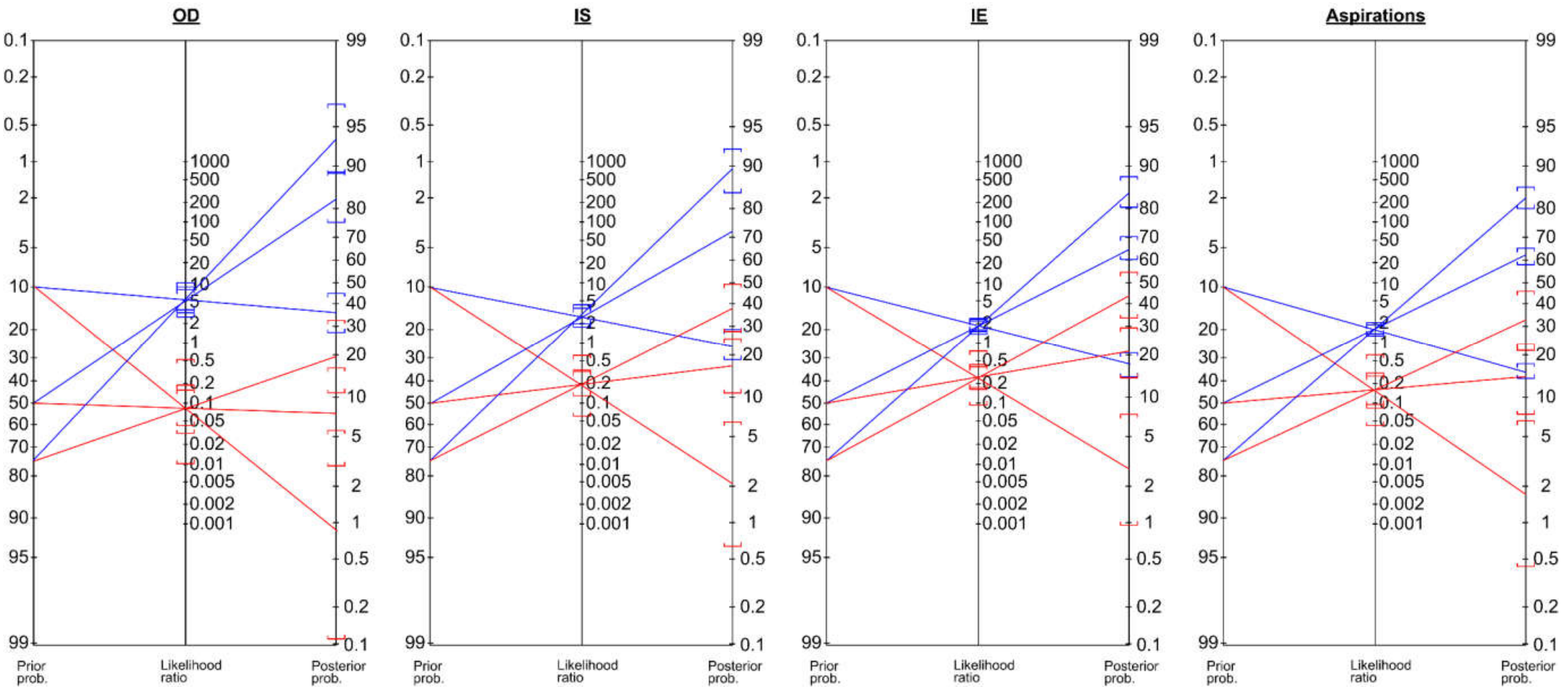

Figure S1: Nomogram for Oropharyngeal Dysphagia (OD), Impaired Safety (IS), Impaired Efficacy (IE) and Aspirations combining the resultant LHR + (in blue) and LHR – (in red) for a Pre-test Prevalence of 10%, 50% and 75%.
