# Supplementary figures and images for: A Systematic and a Scoping Review on the Psychometrics and Clinical Utility of the Volume-Viscosity Swallow Test (V-VST) in the Clinical Screening and Assessment of Oropharyngeal Dysphagia
Source: Foods. 2021 Aug 16;10(8):1900. doi: 10.3390/foods10081900 (PMC8391460; doi:10.3390/foods10081900)

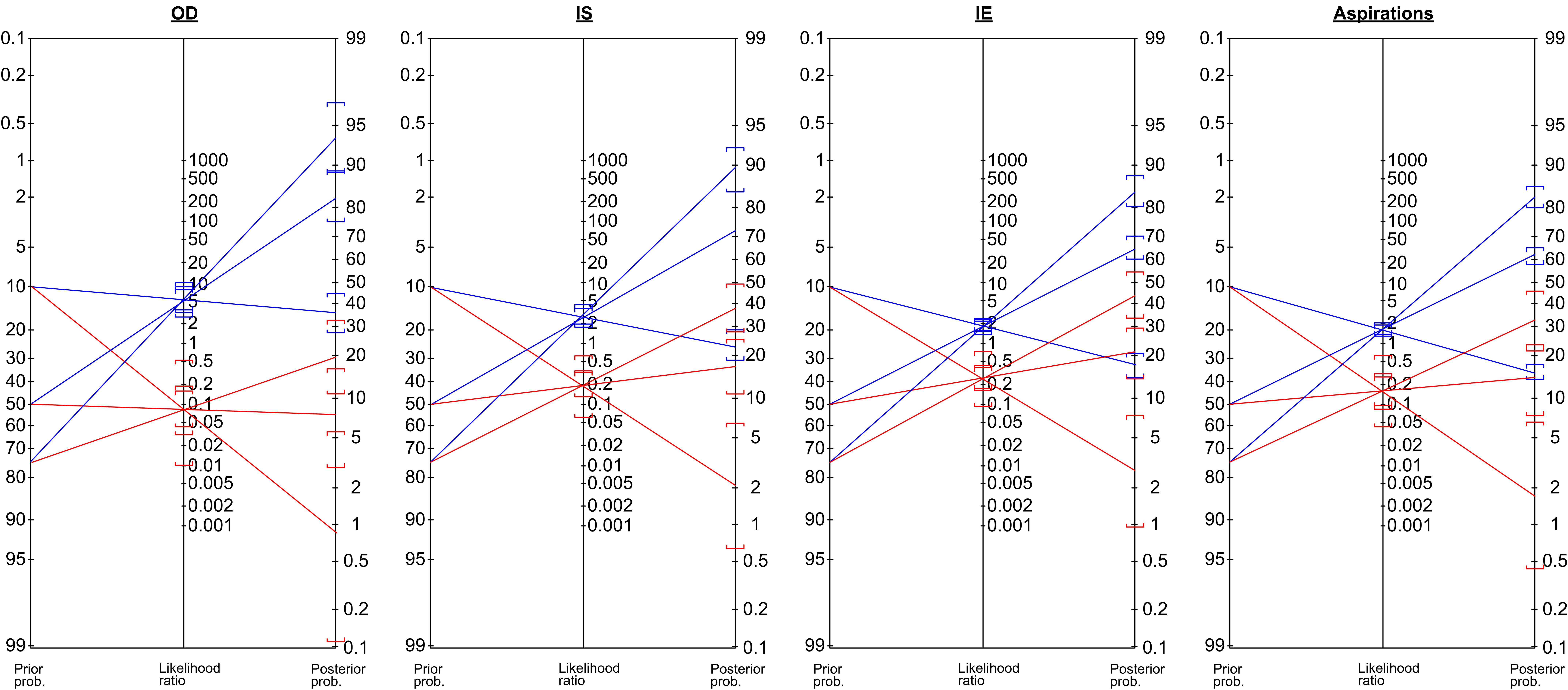

Supplement: Supplementary file 1 [file foods-10-01900-s001.zip › Figure S1.tif]
